# Over Half of Patients Progress to Cartilage Restoration After Initial Cartilage Biopsy in a Military Population

**DOI:** 10.1002/ars2.70064

**Published:** 2026-08-24

**Authors:** Ryan M. Sanborn, Natalia A. Pluta, Kenneth J. Fellows, Christian A. Cruz, Jeanne C. Patzkowski, Daniel J. Cognetti

**Affiliations:** ^1^ School of Medicine Uniformed Services University of the Health Sciences Bethesda Maryland U.S.A.; ^2^ Department of Orthopaedic Surgery Brooke Army Medical Center Fort Sam Houston Texas U.S.A.; ^3^ Department of Orthopaedic Surgery Alexander T. Augusta Military Medical Center Fort Belvoir Virginia U.S.A.; ^4^ United States Army Institute of Surgical Research Fort Sam Houston Texas U.S.A.

## Abstract

**Purpose:**

To evaluate the rate of matrix‐induced autologous chondrocyte implantation (MACI) after cartilage biopsy in a military population.

**Methods:**

A retrospective review was conducted of all patients who underwent a cartilage biopsy at 2 large regional military health centers between January 2021 and May 2023. Patients 17 years of age or older who underwent an index biopsy more than 2 years prior to chart review were eligible for inclusion. The primary outcome was progression from cartilage biopsy to MACI. Secondary outcomes included complication rates and lesion characteristics. Continuous variables were compared using an unpaired t‐test, and categorical variables were analyzed using Fisher's exact test.

**Results:**

A total of 56 patients were included in the study, with a mean age of 31.9 ± 8.3 years. Thirty‐five (62.5%) patients were male, and 50 patients (89%) were current or former military service members. Two patients underwent bilateral knee biopsies, yielding 58 knees, with 32 proceeding to MACI for a 55.2% implantation rate. Multifocal chondral defects were noted in 59% of knees. The most common defect location was the patella (64%). Reoperation occurred in 22% of cases after MACI.

**Conclusions:**

In this military cohort, just over half of patients who underwent an initial cartilage biopsy proceeded to MACI within 2 years.

**Level of Evidence:**

Level IV, therapeutic retrospective case series.

## Graphical abstract

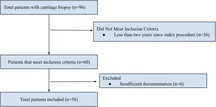

Chondral defects of the knee represent a common orthopaedic concern, particularly among young, active individuals.^1^, ^2^ If left untreated, these defects can progress, leading to significant disability, pain, and osteoarthritis.^3^ Treatment options include chondroplasty, microfracture, osteochondral autograft transfer system, osteochondral allografts, and various autologous chondrocyte implantation procedures, including matrix‐induced autologous chondrocyte implantation (MACI).^4^ Among these, MACI has emerged as a promising strategy, with increasing use in the United States.^4^, ^5^

MACI involves a 2‐stage surgical procedure, with an initial cartilage specimen that is used to seed a scaffold with autologous chondrocytes, which is implanted at the defect site at a later date.^3^, ^6^, ^7^ Studies have shown favorable long‐term outcomes for MACI, including positive patient‐reported outcomes and high satisfaction rates, approaching 90%, at 10 years. Additionally, complete defect fill has been noted in up to 68% of cases with low rates of graft failure, ranging from 2.7% to 9%, at 10 years.^4^, ^7^, ^8^, ^9^ However, not all patients progress to second‐stage implantation. This can be because of a variety of factors, including adequate symptom relief after the initial surgery and financial or insurance‐related considerations, which can be a substantial barrier because of the high cost of the implant.^10^, ^11^ Additionally, because the cartilage procedure itself does not incur an additional cost, it is sometimes performed indiscriminately “to keep options open,” even if the likelihood of eventual implantation is low.

Reflecting these considerations, 2 prior studies have reported low overall implantation rates after biopsy, ranging from 26% to 35% in civilian populations over a minimum of 2 years.^12^, ^13^ These findings, however, may not be generalizable to the military setting, which operates under a single‐payer system and imposes specific occupational demands. Such factors likely influence both the threshold for biopsy and decisions regarding implantation, which define the rate of implantation after biopsy.

The purpose of this study was to evaluate the rate of MACI after cartilage biopsy in a military population. We hypothesized that this rate in the military population would be lower than those observed in civilian settings.

## METHODS

The study was approved by the Brooke Army Medical Center (IRB # C.2025.068e). This retrospective study evaluated patients who underwent a knee cartilage biopsy between January 2021 and May 2023. After Institutional Review Board approval was obtained, medical records were retrospectively reviewed to identify all skeletally mature patients aged 17 years and older who underwent a cartilage biopsy by military orthopaedic surgeons (D.J.C., J.C.P.) at 2 large regional military health centers. Exclusion criteria included insufficient perioperative documentation in the electronic medical record and less than 2 years of follow‐up from the initial biopsy. Prior knee surgery and concomitant procedures did not represent exclusion criteria.

### Indications for Biopsy

Cartilage biopsy was performed at the discretion of the treating surgeon in patients with symptomatic, focal chondral defects that had failed nonoperative management and were considered potentially amenable to cartilage restoration with MACI. Indications for biopsy were based on defect characteristics, including size, severity, location, and osseous involvement, as well as patient‐specific factors such as age, alignment, activity level, and occupational demands.

### Surgical Technique

All cartilage biopsies were performed arthroscopically during an index knee procedure using standard techniques. After diagnostic arthroscopy and treatment of concomitant intra‐articular pathology, chondroplasty of the lesion(s) was performed. Cartilage biopsy specimens were obtained from a nonweight‐bearing region of the knee in accordance with manufacturer recommendations for MACI (Vericel, Cambridge, MA) and were processed per institutional and manufacturer protocols.

For patients who subsequently underwent second‐stage MACI, the procedure was performed using standard open or mini‐open techniques based on defect location and surgeon preference. Prior to implantation, defects were prepared to create a stable rim with the removal of the calcified cartilage layer from the base of the defect, taking care to avoid violation of the subchondral bone.

### Data Collection and Statistical Analysis

Demographic variables collected included age at biopsy, sex, body mass index, active‐duty status, military rank, and prior surgical history. Operative reports from the index biopsies were reviewed for date of biopsy, surgeon, chondral defect location(s), defect size, Outerbridge grade, and concomitant procedures. Likewise, operative reports for patients who underwent subsequent MACI were reviewed to identify the date of implantation, time from biopsy to second‐stage implantation, site of implantation, and associated procedures. Postoperative outcomes included complications (i.e., pain, symptomatic hardware, arthrofibrosis, surgical site infection, and effusion), reoperations, and the impact of knee symptoms on military readiness. More specifically, an additional characterization of military‐specific outcomes was performed, including return to full unrestricted duty, return to duty with permanent activity restrictions, and medical separation because of knee‐related disability.

Continuous variables (i.e., mean defect size) were compared using unpaired t‐test. The associations between categorical variables (i.e., implantation rates of single vs multiple defects) were analyzed using Fisher's exact test. Significance was defined as *P* < .05.

## RESULTS

### Demographics

**FIGURE 1:**
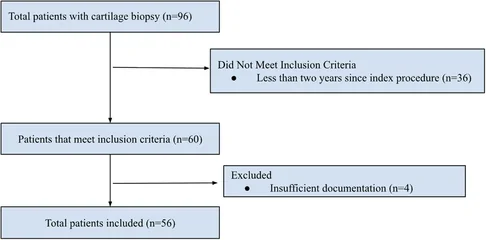
Flow diagram of included and excluded patients.

A total of 56 patients underwent a cartilage biopsy by 15 surgeons at 2 large regional military health centers (Figure 1, Table 1). All patients had a minimum of 2 years from the initial biopsy. The cohort consisted of 35 males (62.5%) and 21 females (37.5%). The mean age of patients was 31.9 ± 8.3 years (range: 17‐56), with an average body mass index of 28.6 ± 3.3 kg/m^2^ (range: 21‐36.8 kg/m^2^). Most patients were current or former military service members, with 62.5% being active‐duty or reservists at the time of initial biopsy and 27% being retirees or separated individuals. Seventy percent of these were enlisted personnel. Two patients underwent biopsies on bilateral knees at separate procedures, resulting in a total of 58 initial biopsies (Table 2).

**TABLE 1:** Baseline Patient Characteristics

	N = 56
Age, y	31.9 ± 8.3
No. of males	35 (62.5)
BMI	28.6 ± 3.3
Military service	
Active duty	31 (56)
Reserve	4 (7)
Retired/separated	15 (27)
Dependent	6 (11)
Military branch	
Army	25 (45)
Navy	3 (5)
Air Force	18 (32)
Marine Corps	3 (5)
Space Force	1 (2)
Military rank	
Enlisted	39 (70)
Warrant officer	2 (4)
Commissioned officer	9 (16)

*Note*: Data presented as mean ± SD or n (%).

BMI, body mass index; SD, standard deviation; y, years.

**TABLE 2:** Patient Characteristics by Chondral Biopsy and MACI Status

	Biopsy (N = 58)	Implanted (N = 32)	Not Implanted (N = 28)
Age, y	32.0 ± 8.3	30.9 ± 8.2	32.9 ± 8.6
No. of males	37 (64)	19 (59)	18 (64)
BMI	28.5 ± 3.2	28.3 ± 3.5	28.6 ± 2.8
Military service			
Active duty	32 (55)	19 (59)	14 (50)
Reserve	4 (7)	1 (3)	3 (11)
Retired/separated	16 (28)	8 (25)	8 (29)
Dependent	6 (10)	4 (13)	3 (11)
Military branch			
Army	26 (45)	14 (44)	13 (46)
Navy	3 (5)	2 (6)	1 (4)
Air Force	19 (33)	12 (38)	7 (25)
Marine Corps	3 (5)	‐	3 (11)
Space Force	1 (2)	‐	1 (4)
Military rank			
Enlisted	41 (71)	24 (75)	17 (61)
Warrant officer	2 (3)	‐	2 (7)
Commissioned officer	9 (16)	4 (13)	6 (21)

*Note*: Data presented as mean ± SD or n (%).

BMI, body mass index; MACI, matrix‐induced autologous chondrocyte implantation; SD, standard deviation; y, years.

Ultimately, 32 MACI implantations (30 patients) were performed after an initial biopsy, for an overall implantation rate of 55.2%. The implantation rate for males was 51% (n = 19), compared with 61.9% (n = 13) for females (*P* = .48). Twenty‐eight (88%) of these procedures were performed on current or former military service members, with 24 (75%) of these being enlisted personnel. The average time from initial biopsy to MACI was 5.8 ± 4.8 months, and there was no significant difference in age (*P* = .36), sex (*P* = .75) or body mass index (*P* = .66) among patients who underwent MACI and those who did not. Notably, 2 patients used the cells from a single biopsy to undergo MACI on both knees.

### Surgical Variables and Outcomes

Most patients had chondral defects at multiple sites within the knee (59%, n = 34), including 18 patients (31%) with bipolar lesions. The most common location of those with only 1 documented site was the patella (58%, n = 14) (Table 3). Rates of implantation among those with multiple and singular defects within the knee were 55.9% (19/34) and 54.2% (13/24), respectively (*P* = .99).

**TABLE 3:** Characteristics of Cartilage Defects

	Biopsy (N = 58)	Implanted (N = 32)	Not Implanted (N = 28)
Primary biopsy location			
LFC	4 (7)	‐	4 (14)
MFC	3 (5)	3 (9)	‐
Patella	14 (24)	8 (25)	7 (25)
Trochlea	3 (5)	2 (6)	1 (4)
Multiple sites	34 (59)	19 (59)	16 (57)
Mean size of largest defect, cm^2^	3.2 ± 1.6	3.5 ± 1.6	2.9 ± 1.6
Severity of largest defect*			
Grade 2	1 (2)	‐	1 (4)
Grade 3	20 (34)	10 (31)	10 (36)
Grade ¾	15 (26)	8 (25)	7 (25)
Grade 4	19 (33)	11 (34)	9 (32)
Unknown	3 (5)	3 (9)	1 (4)

*Note*: Data presented as mean ± SD or n (%).

LFC, lateral femoral condyle; MFC, medial femoral condyle; SD, standard deviation.

* Outerbridge classification.

The mean size of the largest cartilage defect within each knee was 3.2 ± 1.6 cm^2^. No significant difference was noted between the mean size of the largest defects when comparing the implanted and nonimplanted groups (*P* = .13).

Among the 32 patients who underwent MACI, 22 (68.8%) received single‐site implantation, most commonly involving the patella (n = 12, 37.5%), followed by the trochlea (n = 5, 15.6%), medial femoral condyle (MFC) (n = 4, 12.5%), and lateral femoral condyle (n = 1, 3.1%). The remaining 10 patients (31.2%) underwent multisite implantation, including patella and trochlea (n = 6, 18.8%), MFC and lateral femoral condyle (n = 2, 6.3%), patella and MFC (n = 1, 3.1%), and trochlea and MFC (n = 1, 3.1%).

A summary of concomitant procedures performed during the initial biopsy is provided in Table 4. The most common procedures were chondroplasty (n = 45), loose body removal (n = 8), meniscectomy (n = 5), and meniscal repair (n = 3).

**TABLE 4:** Concomitant Procedures Performed During Initial Biopsy

	N = 58
Chondroplasty	45 (78)
Loose body removal	8 (14)
Meniscectomy	5 (9)
Meniscus repair	3 (5)
Plica excision/debridement	2 (3)
PRP injection	2 (3)
Lateral release	1 (2)
Osteochondral defect ORIF	1 (2)
Distal femur osteotomy	1 (2)
Debridement of failed MACI	1 (2)
Trochlear microfracture	1 (2)
Open patella curettage and bone grafting	1 (2)
Patella cyst biopsy	1 (2)
Iliac crest bone marrow biopsy	1 (2)

*Note*: Data presented as n (%).

MACI, matrix‐induced autologous chondrocyte implantation; ORIF, open reduction internal fixation; PRP, platelet‐rich plasma.

Among MACI patients, 15 (47%) underwent a concomitant procedure at the time of implantation. The most frequent was tibial tubercle osteotomy (TTO) (n = 11, 34%), followed by medial patellofemoral ligament (MPFL) or medial quadriceps tendon‐femoral ligament reconstruction (n = 5, 16%) and lateral retinacular lengthening or release (n = 4, 13%). Less common procedures included revision anterior cruciate ligament reconstruction (n = 1, 3%), meniscal repair (n = 1, 3%), loose body removal (n = 1, 3%), hardware removal (n = 1, 3%), chondroplasty (n = 1, 3%), and patellar tendon heterotopic ossification debridement and repair (n = 1, 3%).

The most common postoperative complication after MACI was pain (n = 9, 28%), followed by symptomatic hardware (n = 2, 6%), arthrofibrosis (n = 2, 6%), effusion (n = 1, 3%), and surgical site infection (n = 1, 3%). Reoperation was required in 7 patients (22%) for hardware removal (n = 2), patellar osteochondral allograft transplantation, repeat chondroplasty, lysis of adhesions for arthrofibrosis, repeat MACI with MPFL reconstruction, and irrigation and debridement. Both patients undergoing hardware removal had undergone TTO previously.

### Military‐Specific Outcomes

From a military standpoint, 4 patients (11%, n = 35) who were servicemembers and underwent an initial biopsy proceeded to a medical evaluation board and were ultimately medically separated. This included 2 servicemembers (10%, n = 20) who had received a MACI procedure. Additionally, 4 servicemembers remain on medical profiles, indicating activity restrictions because of injury or illness, including 2 who had a MACI procedure.

## DISCUSSION

Our study shows that within the Military Health System, 55.2% of individuals who underwent an initial cartilage biopsy proceeded to definitive cartilage restoration surgery with MACI. Contrary to the hypothesis, this implantation rate was higher than that previously reported in civilian populations.^12^, ^13^ The reasons for this discrepancy are likely multifactorial.

One important factor is the unique occupational demands of servicemembers. Military personnel must maintain physical fitness standards and engage in regular, vigorous activity, which increases the likelihood of persistent symptoms after chondroplasty and biopsy alone. Although military‐specific factors such as patient or surgeon relocation may interrupt or delay completion of staged procedures, external pressure to maintain readiness and return to unrestricted duty likely favors progression to second‐stage implantation.

In addition to occupational demands, the underlying pathology and frequency of concomitant procedures in this cohort likely influenced the observed implantation rate. Nearly half of patients underwent at least 1 concomitant procedure at the time of biopsy, compared with 13.2% reported in civilian populations, suggesting a greater burden of associated pathology, despite a similar mean age.^14^, ^15^ A substantial proportion of patients also underwent staged TTO and/or MPFL reconstruction at the time of MACI, most of which were preplanned as part of the treatment strategy. A preponderance of these staged realignment procedures may have contributed to the high implantation rate, because correction of underlying patellar instability or malalignment is often required to adequately address the underlying pathologic process. Although patients undergoing staged TTO ± MPFL (n = 11) were excluded, the implantation rate in this cohort remains higher than that reported in prior civilian literature.^12^, ^13^

Defect location further contextualizes these findings, with patellar lesions accounting for nearly 60% of MACI procedures in the present study. Although patellar defects are historically subject to more stringent review by civilian insurers,^11^ the overall distribution of cartilage lesions in our cohort appears comparable to civilian series, with the patella representing the most commonly treated site, followed by the MFC.^12^, ^14^ Taken together, the combination of frequent patellofemoral pathology and a high rate of staged patella stabilizing procedures likely increases the probability of progression to definitive cartilage restoration.

Insurance and payer considerations also likely contribute to the high implantation observed in this cohort. Prior civilian studies have emphasized both the high cost of implantation and restrictive insurer approval criteria as major barriers to MACI. However, for servicemembers within the Military Health System, there are fewer barriers to care and no out‐of‐pocket costs.^10^, ^11^ This distinction is particularly relevant for servicemembers nearing separation or retirement who wish to pursue surgical management while still covered by military insurance. Additionally, whereas most civilian insurers require failure of a prior procedure before approving implantation,^11^ select patients in our cohort proceeded directly to second‐stage MACI using prior contralateral biopsies. While failing a chondroplasty is a reasonable criterion for second‐stage implantation in most cases, in these 2 novel cases, with patients who did well from contralateral MACI, proceeding with definitive management may have been beneficial, because shorter symptom duration prior to implantation has been associated with better outcomes, given that defect expansion occurs at a rate of 0.11 cm^2^ per month of delay.^11^, ^12^, ^14^

Finally, despite a higher upfront cost, multiple studies suggest that MACI may be more cost‐effective than alternatives such as microfracture. Elvidge et al. reported that cartilage restoration reduces the lifetime likelihood of knee replacement by 50% compared with microfracture and is cost‐effective owing to lower overall reoperation rates and improvements in quality of life.^8^, ^16^ Similarly, Basad et al. and Saris et al. found MACI to be significantly more effective than microfracture, particularly for defects ≥3 cm^2^, consistent with the mean defect size of 3.5 cm^2^ in our study population.^6^, ^17^

### Limitations

This study has several limitations. First, the observed MACI implantation rate is likely influenced by heterogeneity in clinical indications and more specifically whether a staged procedure was planned from the outset. For example, in some patients, a biopsy was obtained as part of an anticipated staged approach, such as in the setting of patellar instability requiring concomitant TTO. In others, a biopsy was performed in the setting of an isolated focal chondral defect potentially amenable to chondroplasty alone. Although efforts were made to subcategorize some of these cases, retrospectively distinguishing surgical intent is inherently challenging. Moreover, progression to implantation remains conditional, depending not only on the initial treatment plan but also on postoperative response, functional demands, and other patient‐specific factors.

Second, despite inclusion of 2 large regional military medical centers, the overall sample size remained small, limiting statistical power and precluding meaningful subgroup analyses evaluating the influence of lesion size, location, and other patient‐ and defect‐specific factors on progression to MACI.

Third, although the study was designed to ensure 2 years had elapsed since the cartilage biopsy, chondrocytes can be cryopreserved for up to 5 years. Some patients may undergo implantation beyond the study observation window, potentially resulting in underestimation of the true implantation rate. Finally, while the use of a single electronic health record facilitates longitudinal follow‐up within the Military Health System, outcomes may not be captured once patients separate from service or transition to civilian care.

## CONCLUSIONS

In this military cohort, just over half of patients who underwent an initial cartilage biopsy proceeded to MACI within 2 years.

## DISCLOSURES

The authors (C.A.C., J.C.P., D.J.C.) declare the following financial interests/personal relationships which may be considered as potential competing interests: C.A.C. reports research support from Smith & Nephew. J.C.P. reports a relationship with Arthroscopy Association of North America that includes: board membership; serves on the editorial board for *Arthroscopy: The Journal of Arthroscopic & Related Surgery*. D.J.C. reports a relationship with Arthroscopy Association of North America that includes: board membership; serves on the editorial board for *Arthroscopy: The Journal of Arthroscopic & Related Surgery*. The other authors (R.M.S., N.A.P., K.J.F.) declare that they have no known competing financial interests or personal relationships that could have appeared to influence the work reported in this article.
